# Isomerization of Asp is essential for assembly of amyloid-like fibrils of αA-crystallin-derived peptide

**DOI:** 10.1371/journal.pone.0250277

**Published:** 2021-04-15

**Authors:** Kosuke Magami, Naomi Hachiya, Kazuo Morikawa, Noriko Fujii, Takumi Takata

**Affiliations:** 1 Department of Chemistry, Graduate School of Science, Kyoto University, Sakyo-ku, Kyoto, Japan; 2 Tokyo Metropolitan Industrial Technology Research Institute, Aomi, Koto-ku, Tokyo, Japan; 3 Institute for Integrated Radiation and Nuclear Science, Kyoto University, Kumatori, Sennan-gun, Osaka, Japan; University of Colorado Denver School of Medicine, UNITED STATES

## Abstract

Post-translational modifications are often detected in age-related diseases associated with protein misfolding such as cataracts from aged lenses. One of the major post-translational modifications is the isomerization of aspartate residues (L-isoAsp), which could be non-enzymatically and spontaneously occurring in proteins, resulting in various effects on the structure and function of proteins including short peptides. We have reported that the structure and function of an αA66–80 peptide, corresponding to the 66–80 (^66^SDRDKFVIFLDVKHF^80^) fragment of human lens αA-crystallin, was dramatically altered by the isomerization of aspartate residue (Asp) at position 76. In the current study, we observed amyloid-like fibrils of L-isoAsp containing αA66–80 using electron microscopy. The contribution of each amino acid for the peptide structure was further evaluated by circular dichroism (CD), bis-ANS, and thioflavin T fluorescence using 14 alanine substituents of αA66–80, including L-isoAsp at position 76. CD of 14 alanine substituents demonstrated random coiled structures except for the substituents of positively charged residues. Bis-ANS fluorescence of peptide with substitution of hydrophobic residue with alanine revealed decreased hydrophobicity of the peptide. Thioflavin T fluorescence also showed that the hydrophobicity around Asp76 of the peptide is important for the formation of amyloid-like fibrils. One of the substitutes, H79A (SDRDKFVIFL(L-isoD)VKAF) demonstrated an exact β-sheet structure in CD and highly increased Thioflavin T fluorescence. This phenomenon was inhibited by the addition of protein-L-isoaspartate *O*-methyltransferase (PIMT), which is an enzyme that changes L-isoAsp into Asp. These interactions were observed even after the formation of amyloid-like fibrils. Thus, isomerization of Asp in peptide is key to form fibrils of αA-crystallin-derived peptide, and L-isoAsp on fibrils can be a candidate for disassembling amyloid-like fibrils of αA-crystallin-derived peptides.

## 1. Introduction

**Fig 1:**
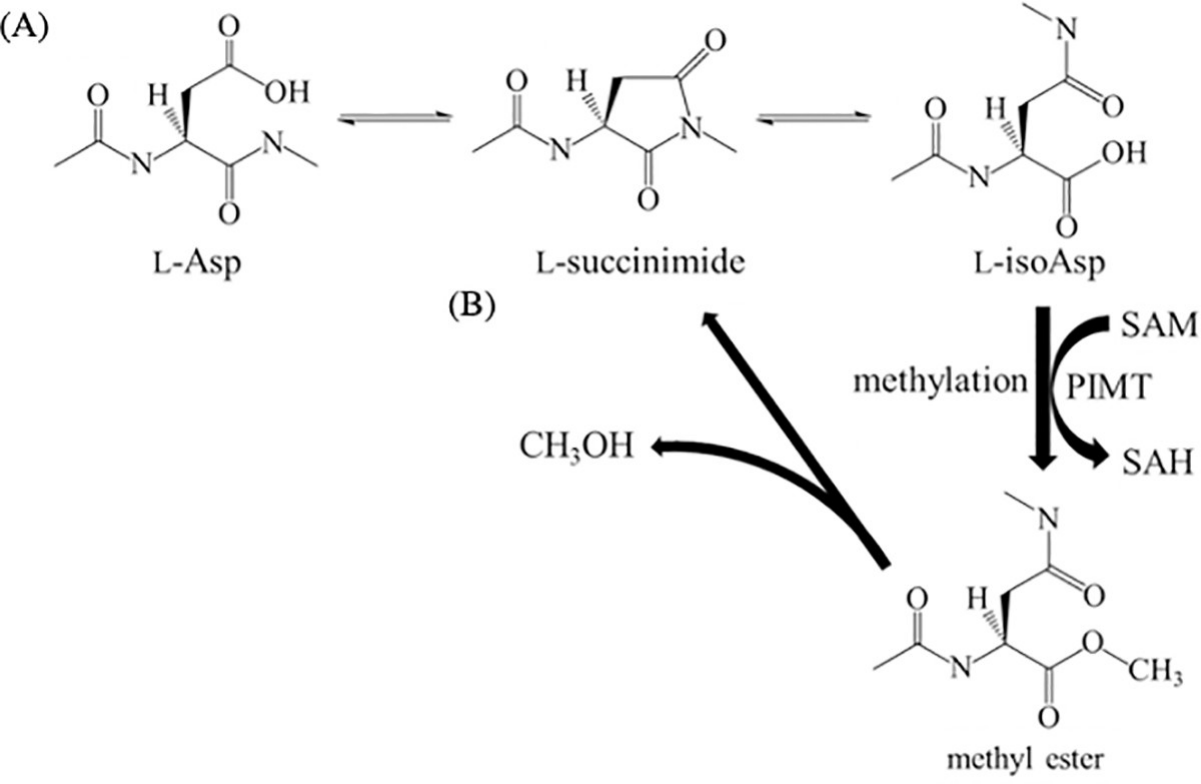
Scheme for isomerization of Asp residues via L-succinimidyl reaction intermediate and repair mechanism of PIMT in protein tertiary structure. (A) Formation of L-Asp isomerization via a five membered-succinimide intermediate in proteins and peptides. (B) L-isoAsp can be converted into L-Asp by the enzyme, protein-L-isoaspartate *O*-methyltransferase (PIMT), through methyl esterification in the presence of SAM. The methyl ester undergoes spontaneous demethylation to form a L-succinimide intermediate. L-succinimide is hydrolyzed to L-isoAsp and L-Asp. The L-isoAsp product can render the methylation/demethylation cycle. SAM; *S*-(5′-adenosyl)-L-methionine and SAH; *S*-(5′-adenosyl)-L-homocysteine.

Technical progress and improved resolution of mass spectrometry (MS) has made it possible to trace tiny differences in chemically modified amino acid residues in proteins. Oxidation, phosphorylation, deamidation, and isomerization of amino acid residues have been identified and quantified by MS [1–4]. Among them, the identification of the L-isoAsp is based on a relatively specific concept of mass shift or different retention times of chromatograms described by the same mass [5–9]. However, contributions of L-isoAsp in proteins have been much obscured because of difficulty in making mimic proteins. The spontaneous and non-enzymatic formation of L-isoAsp, altering the α- to β-linkage of Asp main chain in protein, can occur easily through a five-ring succinimide intermediate in proteins (Fig 1A). Although this intermediate was simultaneously racemized into a D-succinimide intermediate, the hydrolysis of the L-succinimide intermediate proceeded rapidly with a ratio of α- to β-linkage of 3:1, resulting in L-isoAsp as the predominant end product [10,11]. Since the backward reaction from L-isoAsp into the L-succinimide intermediate requires a long time to form a six-ring intermediate, the accumulation of L-isoAsp is unavoidable in proteins [10]. Isomerization of Asp introduces an additional carbon atom in the peptide backbone resulting in the elongation of the protein. Therefore, the formation of L-isoAsp may induce abnormal hydrophilicity/hydrophobicity, structure, and protein functions [12,13].

The predominant L-isoAsp was identified in lens tissues from age-related cataract patients [6]. Age-related cataracts develop due to abnormal aggregation of lens proteins. Soluble, correctly packed, and highly concentrated crystallin family is the main structural protein in the lens and contributes to its transparency. The lens is stable, but is exposed to a variety of intrinsic or extrinsic stresses. The lens fiber cell does not have a turnover function, and hence crystallin undergoes various post-translational modifications during lifetime. Many isomerization sites of lens crystallin have been reported [14]. The αA-crystallin, which is one of the α-crystallin subunits, is a molecular chaperone that suppresses aggregates of other crystallin species [15,16]. Nevertheless, several studies have indicated that the region of αA66–80 (^66^SDRDKFVIFLDVKHF^80^) in αA-crystallin, acts as an “anti-chaperone” that induces protein aggregation [16–18].

We have previously reported the contribution of the formation of L-isoAsp76 to the anti-chaperone function and secondary structure of αA66–80 [13]. These results implied that αA66–80, including L-isoAsp, might form amyloid-like fibrils. In this study, we observed the morphology of amyloid-like fibrils and determined the contribution of each of the amino acids in the peptide. To do this, each amino acid was substituted with alanine in αA66–80, including L-isoAsp, and the ability to form amyloid-like fibrils was evaluated. Although it is believed that isomerization proceeds in one direction, PIMT, which can convert β-linkage of Asp in protein into α-linkage, may possibly stop this phenomenon (Fig 1B) [19–21]. It has been reported that PIMT is widely expressed in various species and tissues, including the eye lens [22,23]. The presence of PIMT in the brain is known to be essential for the survival of mice; however, the role of PIMT in other tissues is not clear [24,25]. Therefore, it is important to investigate the function of PIMT in the eye lens. In this study, we applied this enzyme to one of the L-isoAsp-containing alanine substitutes, which significantly increased the formation of amyloid-like fibrils, and investigated whether the enzyme can act or not on the amyloid-like fibrils.

## 2. Materials and methods

### 2.1. Chemicals

All 9-fluorenylmethyloxycarbonyl (Fmoc)-labeled amino acids and resin-bound amino acids were purchased from Watanabe Kagaku (Hiroshima, Japan). Piperidine for eliminating Fmoc during peptide synthesis was purchased from Tokyo Chemical Industry (Tokyo, Japan). *N*-Methylmorpholine and 1, 2-ethanedithiol were purchased from Nacalai Tesque (Kyoto, Japan). HPLC-grade acetonitrile, *N*, *N*-dimethylformamide, diethyl ether, trifluoroacetic acid, thioflavin T, and 4,4′-dianilino-1,1′-binaphthyl-5,5′-disulfonic acid (Bis-ANS) were purchased from Sigma Aldrich (St. Louis, MO, USA). Recombinant human protein-L-isoaspartate *O*-methyltransferase was purchased from NKMAX (Seongnam-si, Republic of Korea). All other analytical chemicals were purchased from FUJIFILM Wako Pure Chemical Industries (Osaka, Japan).

### 2.2. Peptide synthesis and purification of peptide

Alanine substituents of the αA66–80 peptide, corresponding to 66–80 (^66^SDRDKFVIFLDVKHF^80^), including L-isoAsp76, were synthesized by automated Fmoc-based solid-phase peptide synthesis (PSSM-8; Shimadzu, Japan). Fmoc-L-aspartic acid beta-tert-butyl ester (Fmoc-L-Asp (OtBu)-OH) was used to produce Asp, and was converted to Fmoc-L-Asp-OtBu-OH to produce L-isoAsp. The synthesis was performed in *N*, *N*-dimethylformamide (DMF) and contained each Fmoc amino acid (10 equiv.), (benzotriazol-1-yloxy)-tripyrrolidinophosphonium hexafluorophosphate (10 equiv.), 1-hydroxybenzotriazole hydrate (10 equiv.), and *N*-methylmorpholine (7.5 equiv.). Deblocking of the N-terminal Fmoc group was performed with 30% piperidine in DMF. The resulting peptides were cleaved from the resin and the protective groups were removed by incubation for 8 h with a solution of 82.5% trifluoroacetic acid (TFA), 5% water, 5% thioanisole, 3% ethylmethylsulfide, 2.5% 1, 2-ethandithiol, and 2% thiophenol. The crude peptides were purified by reverse-phase (RP)-HPLC using a C18 column (Capcell Pak C18 ACR, 10 × 250 mm^2^; Shiseido, Japan). In the case of mutant αA66–80 peptides, a linear gradient of 25–40% acetonitrile containing 0.1% TFA was applied at a flow rate of 3.0 mL/min with detection at 230 nm. The purity of each peptide was confirmed by RP-HPLC. To confirm the molar mass of each peptide, each sample was directly loaded into an infusion ion trap system (LCQ Fleet, Thermo Fisher Scientific Inc., USA) (S1 Table).

### 2.3. Identification of PIMT activity for L-isoAsp residue

The iso-Asp residue in αA66–80 was confirmed by PIMT treatment and nanoscale reverse phase high performance liquid chromatography (Paradigm MS4, Michrom Bioresources, Auburn, CA) using a C18 column (L-column, 0.1 × 150 mm; Chemicals Evaluation and Research Institute, Tokyo, Japan). The αA66–80 (L-Asp76) and αA66–80 (L-isoAsp76) solutions were prepared at concentrations of 0.1 mg/mL. PIMT (0.02 mg/mL) was added to αA66–80 (L-isoAsp76), and then incubated for 24 h at 37°C. Each peptide (5 μg) was injected into the column and its elution profile was observed. A linear gradient of 5%–45% acetonitrile containing 0.1% formic acid was applied at a flow rate of 0.5 μL/min over 60 min. MS and MS/MS scan data were collected in positive mode with an ion trap system (LCQ Fleet, Thermo Fisher Scientific Inc., USA).

### 2.4. Transmission Electron Microscope (TEM)

The sample solution was prepared as previously described [13]. Two milligrams of peptides were dissolved in 1 mL of distilled water and diluted with 50 mM phosphate buffer (pH 7.4). The final concentration of each peptide was 0.1 mg/mL. The formation of fibrils was detected with a Titan electron microscope (Thermo Fisher Scientific Inc., USA). The samples for imaging were placed on a 300-mesh copper grid and negatively stained with potassium Na-encapsulated Preyssler-type phosphotungstate.

### 2.5. Circular dichroism measurement

Far-UV circular dichroism (CD) was performed using a J-805 instrument (JASCO, Tokyo, Japan). Each αA66–80 peptide (0.1 mg) was dissolved in 1 mL of 10 mM phosphate buffer (pH 7.4). The solution was then diluted with distilled water to 16.6 mM phosphate. CD spectra were acquired in the range of 195–250 nm using a 1 mm path cylindrical quartz cell. All spectra were baseline corrected by subtracting the buffer-only spectrum. Each reported spectrum represents an average of five scans.

### 2.6. Bis-ANS spectroscopic assay

Bis-ANS assay was carried out using a Hitachi F-4500 fluorescence spectrophotometer with excitation at 360 nm and emission at 400–600 nm (Hitachi, Tokyo, Japan). A 20 μM Bis-ANS solution was prepared in 10 mM Tris buffer (pH 7.4). Next, 25 μL of each αA66–80 peptide solution (1.0 mg/mL) was added to 225 μL of Bis-ANS solution. After incubation for 10 s at room temperature, Bis-ANS fluorescence was measured. Each assay was conducted three times.

### 2.7. Thioflavin T spectroscopic assay

Thioflavin T assay was carried out using a Hitachi F-4500 fluorescence spectrophotometer with excitation at 450 nm and emission at 460–600 nm (Hitachi, Tokyo, Japan). A 50 μM Thioflavin T (ThT) solution was prepared in 10 mM phosphate buffer (pH 7.4). Next, 20 μL of each αA66–80 peptide solution (0.3 mg/mL) was added to 250 μL of ThT solution. After incubation for 60 s at room temperature, ThT fluorescence was measured. Each assay was conducted five times.

### 2.8. Measurement of PIMT activity for amyloid fibril peptide

The protein-L-isoaspartate *O*-methyltransferase (PIMT) activity for the peptide was assessed by measuring the intensity of the ThT fluorescence signal using a Hitachi F-4500 fluorescence spectrophotometer with excitation at 450 nm and emission at 460–600 nm. ThT solution (50 μM) was prepared in 10 mM Tris buffer (pH 7.8). Each peptide (0.01 mg/mL) was incubated in 10 mM Tris buffer (pH 7.8) at 37°C for 24 h with PIMT at peptide to PIMT ratios of 1:1, 1:2, and 1:5 (w/w). The amount of *S*-(5′-adenosyl)-L-methionine (SAM) was the same as that of PIMT. Fifty microliter of the sample solution was mixed with 150 μL of ThT solution. After incubation for 60 s at room temperature, ThT fluorescence was measured. Each ThT assay was conducted five times.

## 3. Results

### 3.1. Synthesis and electron microscopy of αA66–80 (L-isoAsp76)

**Fig 2:**
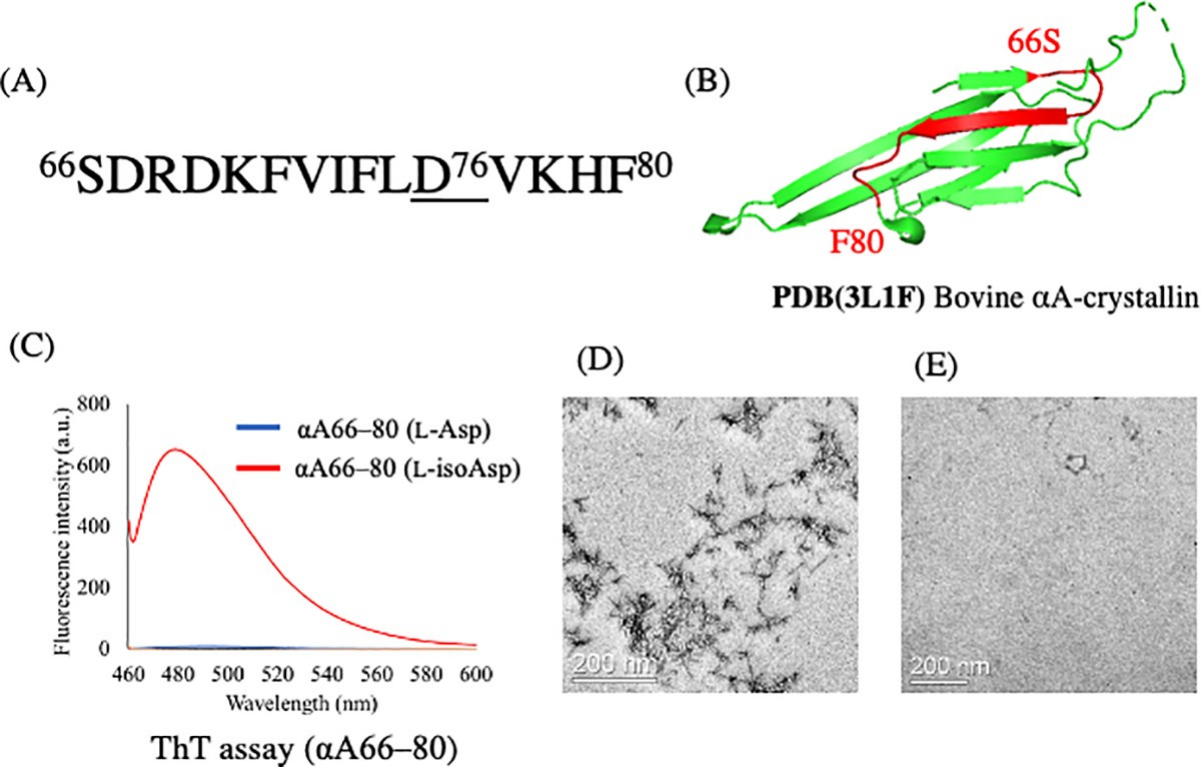
L-isoAsp 76 containing human αA-crystallin 66–80 forms amyloid like fibrils. (A) αA66–80 peptide sequence. The Asp residues at 76 position in the sequence was replaced with the L-isoAsp residue. (B) The 3D structure of bovine αA-crystallin (PDB: 3L1F). The αA66–80 region of human αA-crystallin sequence is colored red in the structure. (C) Fluorescence spectra of ThT in the presence of L-Asp in the peptide after heat incubation (blue) and the amyloid fibrils formed from L-isoAsp-containing peptide (red). (D) TEM images of negatively stained fibrils of L-isoAsp containing αA66–80 after heat incubation. (E) TEM images of negatively stained fibrils of L-Asp containing αA66–80 after heat incubation.

Fig 2A shows the sequence of αA66–80 with the position of Asp76 in human lens αA-crystallin. It is located on the predicted β-sheet region of homologous bovine αA-crystallin (Fig 2B). This synthetic peptide containing Asp at the 76^th^ position was replaced with L-isoAsp and showed relatively intense ThT fluorescence, implying the formation of amyloid-like fibrils (Fig 2C). The L-isoAsp in the peptide could be enzymatically changed to L-Asp after PIMT treatment (S1 Fig). The TEM image of this peptide clearly showed the presence of short length rod-like structures of amyloid-like fibrils (Fig 2D). These findings were not observed in the case of L-Asp-containing peptide (Fig 2E). These data suggested that the isomerization of Asp76 in αA66–80 induced short length amyloid-like fibril formation.

### 3.2. Secondary structure of alanine substituents αA66–80 (L-isoAsp76)

**Fig 3:**
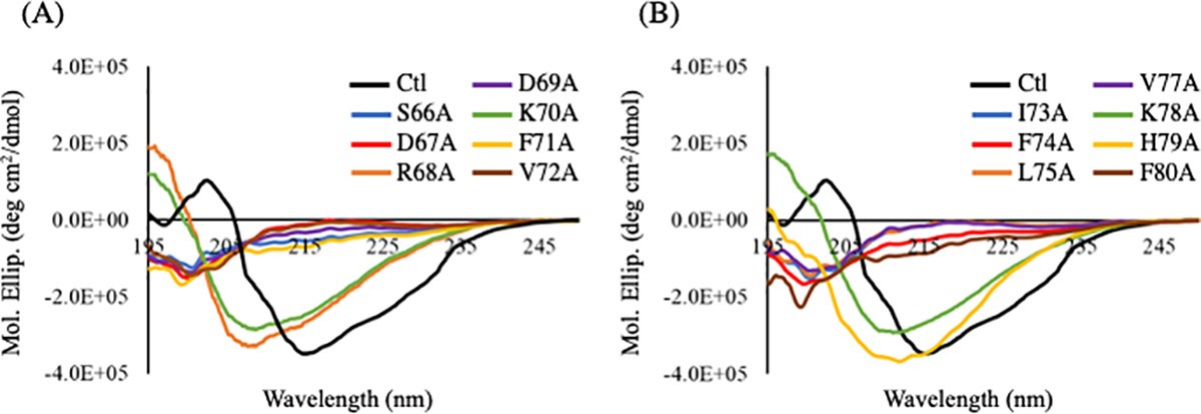
Far-UV CD spectra of the alanine substituents of αA66–80 (L-isoAsp76) peptides. Black line shows the far-UV CD spectrum of the control αA66–80 (L-isoAsp76: Ctl) peptide. (A) Blue, red, orange, purple, green, yellow, and brown lines show the far-UV CD spectra of S66A, D67A, R68A, D69A, K70A, F71A, and V72A substituents, respectively. (B) Blue, red, orange, purple, green, yellow, and brown lines show the far-UV CD spectra of I73A, F74A, L75A, V77A, K78A, H79A, and F80A substituents, respectively. The spectra were recorded between 195 and 250 nm. Each spectrum represents an average of five scans.

To screen the specific interactions between L-isoAsp and other amino acids, individual alanine substitution was performed for each residue of the peptide. The alanine substituents of αA66–80 peptide, including L-isoAsp76, were synthesized and purified in the same manner as αA66–80. The sequences of the substituents are shown in S1 Table. To confirm whether each peptide was synthesized correctly, analytical LC-MS was performed. The theoretical and actual masses of the protonated precursor ions of the synthetic peptides are shown in S1 Table. The purity of the synthetic peptides was confirmed to be >95%. Fig 3 shows the CD spectra of the alanine substituents of αA66–80 (L-isoAsp76). In CD spectra of R68A, K70A, K78A, and H79A substituents of αA66–80 (L-isoAsp76), we observed that the β-sheet structures were retained. However, the other alanine substituents of αA66–80 peptides did not form a β-sheet structure. These results indicated that the positively charged amino acids were not important for forming the β-sheet structure of αA66–80 (L-isoAsp76).

### 3.3. The alanine substituent changes the hydrophobicity of αA66–80 (L-isoAsp76)

**Fig 4:**
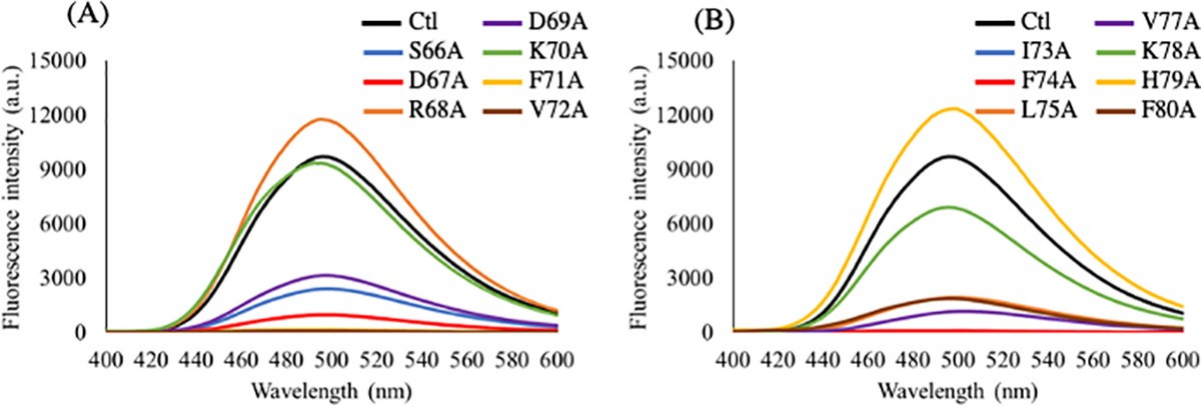
Emission spectra of Bis-ANS solution in the presence of the alanine substituents of αA66–80 peptides. Black lines show the emission spectrum of the Bis-ANS solution in the presence of normal αA66–80 (L-isoAsp76: Ctl). (A) Blue, red, orange, purple, green, yellow, and brown lines show the emission spectra of the Bis-ANS solution in the presence of S66A, D67A, R68A, D69A, K70A, F71A, and V72A substituents, respectively. (B) Blue, red, orange, purple, green, yellow, and brown lines show the emission spectra of the Bis-ANS solution in the presence of I73A, F74A, L75A, V77A, K78A, H79A, and F80A substituents, respectively. Each spectrum represents an average of three scans.

Bis-ANS shows the increasing fluorescence intensity when binding to multiple sites through hydrophobic interactions. Therefore, the change of hydrophobicity of a peptide can be estimated by measuring the intensity of Bis-ANS fluorescence. In this experiment, the R68A, K70A, K78A, and H79A substituents of αA66–80 (L-isoAsp76) did not decrease the Bis-ANS fluorescence intensity much (Fig 4). The other alanine substituents of αA66–80 (L-isoAsp76) showed a reduction in the Bis-ANS fluorescence intensity, suggesting that alanine substitution except for positively charged amino acids decreases the hydrophobicity of αA66–80 (L-isoAsp76). The results were consistent with the CD spectra, suggesting that the change in secondary structure is caused by the change in hydrophobicity.

### 3.4. One of the alanine substituents, H79A, is most likely to form amyloid-like fibrils

**Fig 5:**
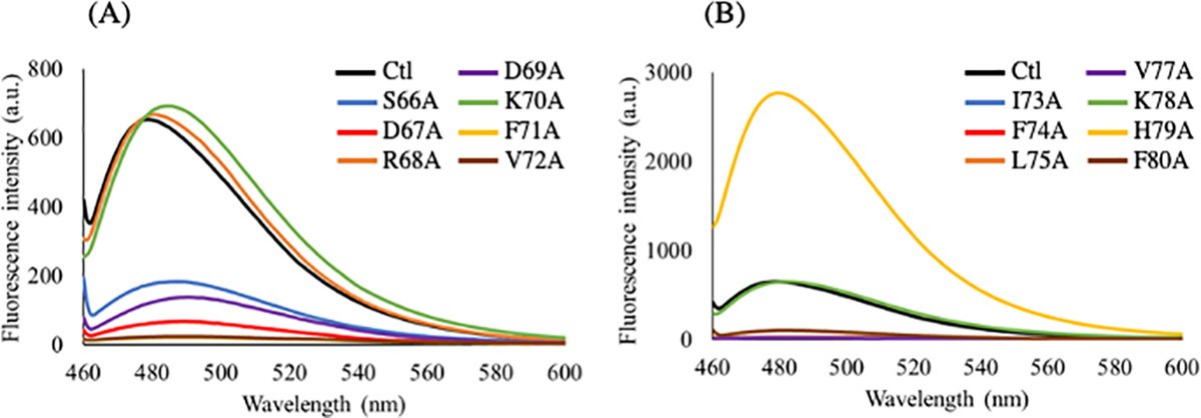
Emission spectra of the Thioflavin T (ThT) solution in the presence of the alanine substituents of αA66–80 peptides. Black lines show the emission spectrum of the ThT solution in the presence of normal αA66–80 (L-isoAsp76: Ctl). (A) Blue, red, orange, purple, green, yellow, and brown lines show the emission spectra of the ThT solution in the presence of S66A, D67A, R68A, D69A, K70A, F71A, and V72A substituents, respectively. (B) Blue, red, orange, purple, green, yellow, and brown lines show the emission spectra of the ThT solution in the presence of I73A, F74A, L75A, V77A, K78A, H79A, and F80A substituents, respectively. Each spectrum represents an average of five scans.

Thioflavin T (ThT) shows specific binding to the amyloid state of the protein, and the change in ThT fluorescence intensity could be used to characterize the formation of amyloid fibrils. In this study, the ThT assay was carried out to determine whether alanine substituents of αA66–80 (L-isoAsp76) could form amyloid-like fibrils (Fig 5). Under the current experimental conditions, the R68A, K70A, and K78A substituents of αA66–80 (L-isoAsp76) did not alter the fluorescence intensity as αA66–80 (L-isoAsp76). The H79A substituent of αA66–80 (L-isoAsp76) showed highly intense fluorescence emission, suggesting that the H79A substituent of αA66–80 (L-isoAsp76) is more likely to form amyloid-like fibrils (Fig 5B). The H79A substituent of αA66–80 (L-Asp76) showed a little increase in the ThT fluorescence intensity (S2 Fig), indicating that it can form small amounts of amyloid-like fibrils. These results also suggest that the alanine substitution for histidine residue promotes the formation of amyloid-like fibrils of αA66–80 (L-isoAsp76) rather than of αA66–80 (L-Asp76). The negatively charged substituents, D67A and D69A, showed weak fluorescence similar to that of substituents of S66A of αA66–80 (L-isoAsp76) (Fig 5A). The hydrophobic residues decreased the fluorescence emission intensity of ThT at a wavelength near 480 nm. These results showed that R68A, K70A, K78A, and H79A mutant αA66–80 (L-isoAsp76) peptide generated amyloid-like fibrils, consistent with the results of CD analysis.

### 3.5. PIMT acted on the L-isoAsp in the substituent of H79A amyloid-like fibrils

**Fig 6:**
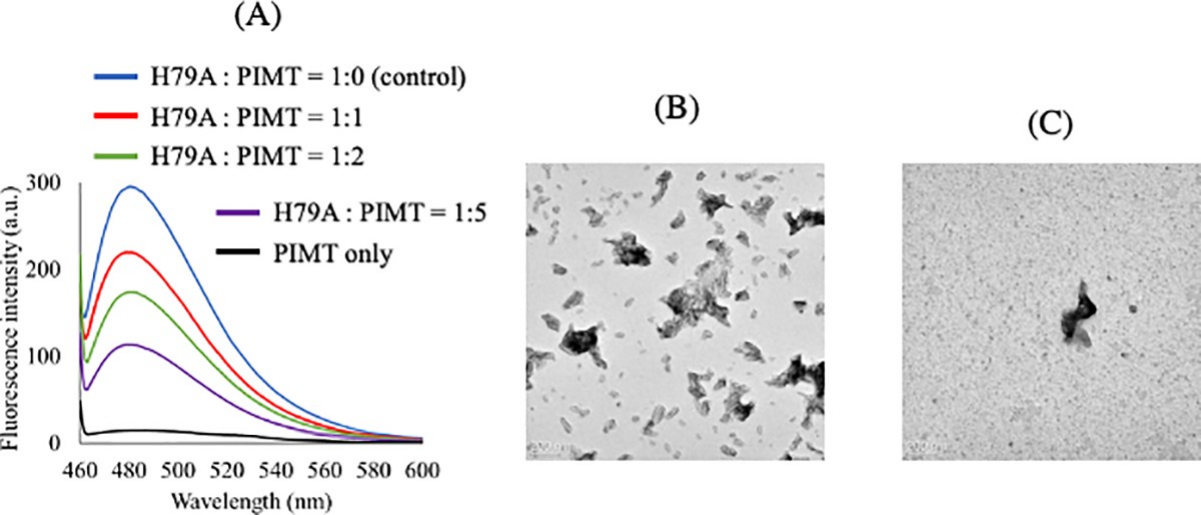
PIMT decreased the formation of amyloid-like fibrils of H79A alanine substituent of αA66–80 peptides. (A) Repair activity of PIMT was monitored as the intensity of ThT fluorescence. The mass ratio of H79A to PIMT was 1:0 (blue), 1:1 (red), 1:2 (green), and 1:5 (purple). Black lines show the emission spectrum of the ThT solution in the absence of peptide. Each spectrum represents an average of five scans. (B) TEM images of negatively stained fibrils of L-isoAsp containing H79A αA66–80 after heat incubation in the absence of PIMT. (C) TEM images of negatively stained fibrils of L-isoAsp containing H79A αA66–80 after heat incubation in the presence of PIMT.

The fluorescence intensity of ThT when only using αA66–80 (L-isoAsp76) was too small to evaluate the PIMT activity. Therefore, we used the abovementioned H79A substituent of αA66–80 (L-isoAsp76), which forms large amounts of amyloid-like fibrils. After heat-induced amyloid formation of H79A peptide, excess amounts of PIMT and SAM were added to induce the backward reaction from L-isoAsp to L-Asp on amyloid-like fibrils. As shown in Fig 6A, the fluorescence emission intensities of ThT decreased according to the concentration of PIMT. TEM image supported the decreased formation of amyloid-like fibrils of the H79A substituent of αA66–80 (L-isoAsp76) after PIMT treatment (Fig 6B and 6C). The results indicated that PIMT could act on L-isoAsp on amyloid-like fibrils and convert it into Asp, thereby, reforming the non-amyloid-like structure of the peptide.

## 4. Discussion

TEM images showed the presence of amyloid-like fibrils (Fig 2D). Short and sharp shapes possibly indicate the seeds of long-length amyloid-like fibril formation, the short shape formed due to low concentrations of peptide (~0.3 mg/mL). Since the morphology of amyloid-like fibrils depends on the concentrations of peptide, an increase in the concentrations of peptide including L-isoAsp corresponded to an increase in extensions observed. However, in TEM images of amyloid-beta 15–28 peptide containing L-isoAsp at the same concentration and pH, we observed shorter structures than those observed in other reports [26]. Therefore, αA66–80 (L-isoAsp76) functions in the formation of amyloid-like fibrils, but the assembly of fibrils would be relatively shorter than that of other peptides that form extended structures.

The current data also suggested that the hydrophilicity of peptides is important for fibril formation. The alanine substituent for positively charged residues did not alter the formation of secondary structure, hydrophobicity, and amyloid-like fibrils (Figs 3–5). Although the side chain of L-isoAsp has a negative charge and interacts with positively charged residues, the data indicated that there was no interaction between L-isoAsp and positively charged residues in amyloid-like fibrils. Nevertheless, it has been suggested that the isomerization of Asp residue at position 76 of αA66–80 is essential for the formation of secondary structure and amyloid-like fibrils (S3 Fig). This implied the significance of L-isoAsp at position 76 of this peptide. These also emphasized the significance of the hydrophobic amino acid network for the formation of amyloid-like fibrils. Previously, the region from F71–V77 residues of αA66–80 has been reported as the core region of the amyloid-forming αA66–80 [27]. Asp76 is located in the core region and the isomerization from Asp to abnormal L-isoAsp would add a carbon in the peptide main chain, thereby altering the hydrophobicity. This may also be a factor in the formation of β-sheet structures as indicated by CD analysis, resulting into amyloid-like fibrils. In a previous study, however, D-isoAsp, involving both isomerization and racemization, did not induce a β-sheet-rich structure [13]. Thus, there may be reasons other than hydrophobicity, that lead to the formation of short amyloid-like fibrils.

The enzymatic treatment using PIMT decreased amyloid-like fibrils of L-isoAsp containing H79A peptide (Fig 6). This treatment was performed after the formation of amyloid-like fibrils; thus, PIMT could recognize L-isoAsp on the surface of the short amyloid-like fibrils, inhibiting fibril-fibril interaction, or loosening fibrils in each unit. As shown above, presumably, the main chain of L-isoAsp76 works as a part of the hydrophobic network in amyloid-like fibrils, but the exposed side chain of L-isoAsp was recognized by PIMT. Here, the concentration of PIMT was higher than that of the peptide. Thus, bulky PIMT inhibition inhibits further extension of amyloid-like fibrils. It is still unclear whether L-isoAsp76 on the H79A mutant αA66–80 (L-isoAsp76) was converted into L-Asp via a five-ring succinimide intermediate. Further studies are needed to confirm the details of the reaction and generality for other peptides, including L-isoAsp. However, as far as we know, this is the first report that PIMT can function as a degrading enzyme for amyloid-like fibrils *in vitro*.

*In vivo*, the presence of peptide αA66–80 and isomerization of Asp76 was confirmed in aged lenses [6,18]. The anti-chaperone function of the peptide was altered; however, the difference in the percentage of isomerization between those in water-soluble fractions and in the water-insoluble fractions was very similar (20% in lens insoluble fractions and 15% in lens soluble fractions) [6,13]. In addition, although the anti-chaperone function seems to be general, its ability was not high. These results suggested that the isomerization of Asp76 did not induce insolubilization of αA-crystallin. Our current data add a new possibility for the αA66–80 (L-isoAsp76) structure *in vivo*. An increase in isomerization should correspond to an increased solubility, but simultaneously forming β-sheet structure and amyloid-like fibrils [13]. The amyloid-like fibrils have never been reported earlier; thus, it is hard to digest by the conventional enzymes for MS analysis. In order to completely quantify the truncated peptide with modification in aged lens, other high-resolution systems with harsh enzymatic methodology would be required. To resolve these problems, a multiple reaction monitoring system with effective enzyme such as PIMT, which was shown in this study, can be applied for quantifying L-isoAsp-containing truncated products in aged lenses [28].

It should be discussed whether the isomerization of Asp76 occurs before or after truncation. Isomerization ratio has been reported to be faster in proteins than in random coiled structures [29]. In that report, authors suggested that a specific field to induce L-succinimide intermediate could be introduced depending on the protein’s tertiary structure. αA66–80 is located near the center of αA-crystallin, which is an important region for the chaperone-like function of αA-crystallin, thus predicting the interface between crystallin families [16,30]. This possibly suggests that isomerization occurs before truncation. To determine this, a comparison of the ratio of Asp isomerization in the peptide and structure can be done in the future. The shorter core region is a better chaperone; therefore, the charged region (on both sides) of αA66–80 would work in a different way from the core region of the peptide.

*In vivo*, the isomerization of Asp76 functions to acquire the chaperone-like activity of αA66–80 (L-isoAsp76) to suppress the aggregation of other lens crystallins. Therefore, the presence of PIMT in lens would be unsuitable. In actuality, the expression levels of PIMT are high in the brain, but low in lens [23]. The physiological role of PIMT has not yet been elucidated. However, the present experiments have shown the potential role of PIMT in loosening amyloid-like fibrils, if they have accessible L-isoAsp on the surface.

## Supporting information

S1 Fig LC-MS chromatogram of the αA66–80 peptide containing L-isoAsp76. (A) LC spectrum of peptide, with a molar mass of 623.0, that eluted from αA66–80 (L-isoAsp76). (B) LC spectrum of peptide, with a molar mass of 623.0, that eluted from αA66–80 (L-Asp76). (C) LC-MS chromatogram of the PIMT-treated αA66–80 (L-isoAsp76). There were two peaks in the LC spectrum on (C). The late elution peak had the same elution time as αA66–80 (L-Asp76). The early elution peak did not show the identical elution time from both, but showed 14 Da mass difference from αA66–80 (L-Asp76) and αA66–80 (L-isoAsp76). This mass difference was derived from methyl group during PIMT interaction with αA66–80 (L-isoAsp76). These showed the activity of PIMT for L-isoAsp76 containing peptide in current experiments.
(TIFF)

S2 Fig Emission spectra of ThT solution in the presence of the H79A αA66–80 (L-Asp76) and H79A αA66–80 (L-isoAsp76) peptides. (TIFF)

S3 Fig Comparison of the secondary structure of αA66–80 (L-isoAsp76) and D76A αA66–80 peptides. (A) Far-UV CD spectra of αA66–80 (L-isoAsp76) (black) and D76A αA66–80 (red). (B) The fluorescence spectra of ThT in the presence of αA66–80 (L-isoAsp76) (black) and D76A αA66–80 (red).
(TIFF)

S1 Table Molar masses of each alanine substituents. (TIFF)
