# Supplementary figures and images for: Isomerization of Asp is essential for assembly of amyloid-like fibrils of αA-crystallin-derived peptide
Source: PLoS One. 2021 Apr 15;16(4):e0250277. doi: 10.1371/journal.pone.0250277 (PMC8049310; doi:10.1371/journal.pone.0250277)

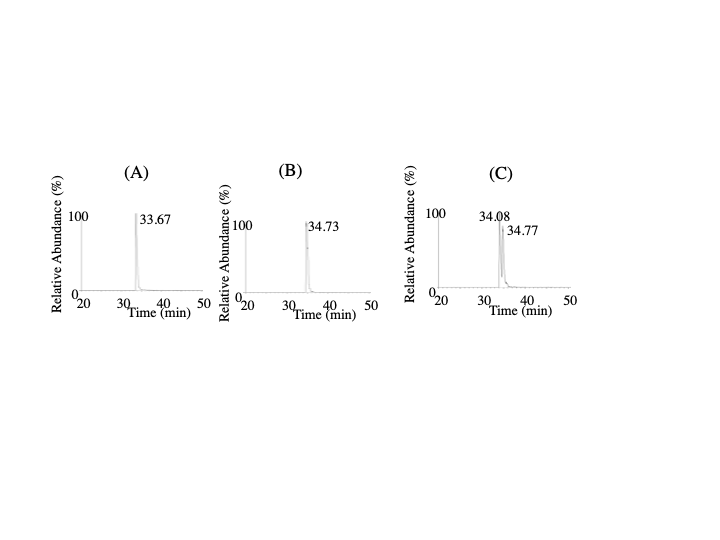

Supplement: S1 Fig — (A) LC spectrum of peptide, with a molar mass of 623.0, that eluted from αA66–80 (L-isoAsp76). (B) LC spectrum of peptide, with a molar mass of 623.0, that eluted from αA66–80 (L-Asp76). (C) LC-MS chromatogram of the PIMT-treated αA66–80 (L-isoAsp76). There were two peaks in the LC spectrum on (C). The late elution peak had the same elution time as αA66–80 (L-Asp76). The early elution peak did not show the identical elution time from both, but showed 14 Da mass difference from αA66–80 (L-Asp76) and αA66–80 (L-isoAsp76). This mass difference was derived from methyl group during PIMT interaction with αA66–80 (L-isoAsp76). These showed the activity of PIMT for L-isoAsp76 containing peptide in current experiments. (TIFF) [file pone.0250277.s001.tiff]

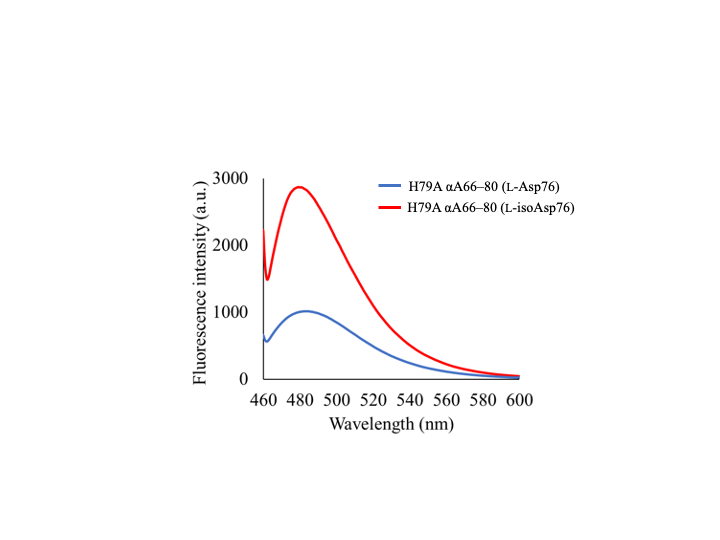

Supplement: S2 Fig — (TIFF) [file pone.0250277.s002.tiff]

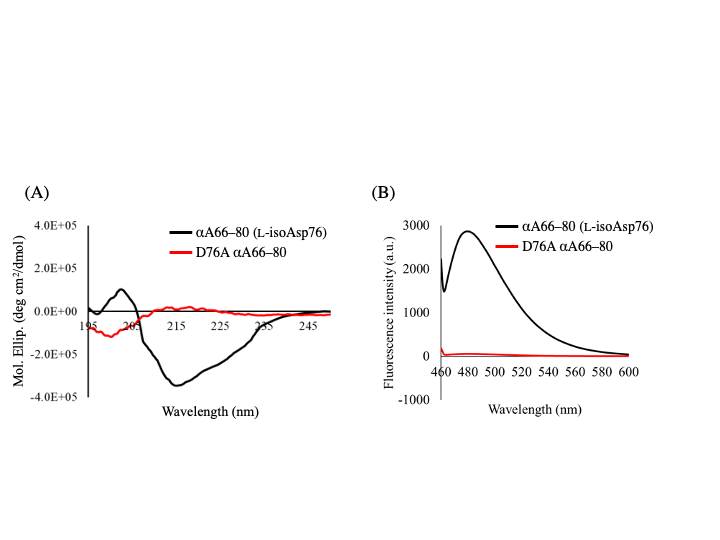

Supplement: S3 Fig — (A) Far-UV CD spectra of αA66–80 (L-isoAsp76) (black) and D76A αA66–80 (red). (B) The fluorescence spectra of ThT in the presence of αA66–80 (L-isoAsp76) (black) and D76A αA66–80 (red). (TIFF) [file pone.0250277.s003.tiff]

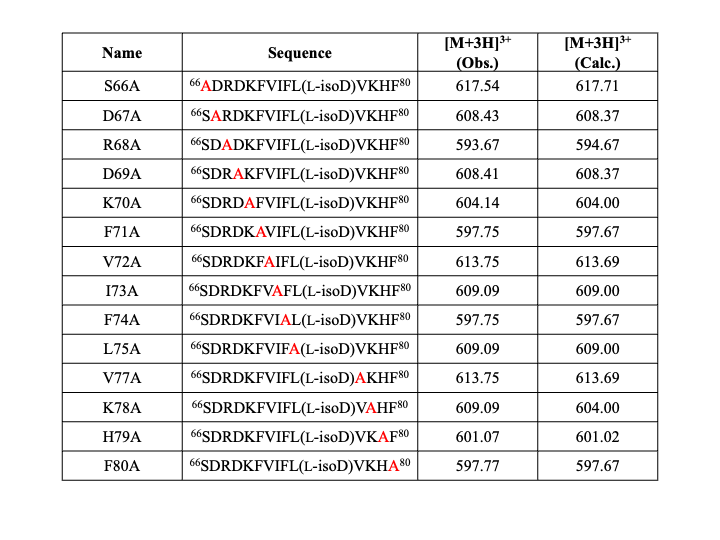

Supplement: S1 Table — (TIFF) [file pone.0250277.s004.tiff]
